# Defining pain-validation: The importance of validation in reducing the stresses of chronic pain

**DOI:** 10.3389/fpain.2022.884335

**Published:** 2022-10-14

**Authors:** Melinda Nicola, Helen Correia, Graeme Ditchburn, Peter D. Drummond

**Affiliations:** Discipline of Psychology, College of Science, Health, Engineering, and Education, Murdoch University, Perth, WA, Australia

**Keywords:** chronic pain, pain-validation, invalidation, stress, pain management, emotional processing

## Abstract

**Purpose:**

To validate an individual's feelings or behaviour is to sanction their thoughts or actions as worthy of social acceptance and support. In contrast, rejection of the individual's communicated experience indicates a denial of social acceptance, representing a potential survival threat. Pain-invalidation, though ill-defined, appears to be a fundamental component of psychosocial stress for people with chronic pain. As such, the aim of this paper was to define pain-validation and outline its importance for those with chronic pain.

**Methods:**

The pain-validation construct was defined using themes inherent in the narratives of those with chronic pain, as identified in a previously published systematic search and thematic analysis, together with examination of additional literature on pain-validation in the clinical context.

**Results:**

We present a construct definition, proposing that pain-validation must necessarily include: (i) *belief* that the pain experience is true for the individual, (ii) *acceptability* of the individual's expressions of pain, and (iii) *communication* of belief and acceptability to the individual experiencing pain. Further, we outline the importance of pain-validation as a protective factor and means of reducing many of the psychosocial stresses of chronic pain; for example, by indicating social support for pain-coping, buffering negative emotions, and re-enforcing unity and shared identity.

**Implications:**

The role of pain-validation in the current era of pain management intervention is discussed. Adhering to interventions that involve cognitive and behavioural change is often difficult. Acknowledging and validating the acceptability of the patient's pain experience in the early stages of pain management may, therefore, be a key component of intervention that encourages compliance to the treatment plan and achieving therapeutic goals.

## Introduction

The need to feel validated can be understood from an evolutionary perspective. As social animals, humans prefer to live in groups to gain survival advantages (1) through protection from threats, sharing of resources and skills, and division of labour (2). Group members who do not fit in with acceptable norms risk being cast out of the group and no longer afforded its protection or access to shared resources necessary for survival. Indeed, the basic need for group belonging and acceptance, and its centrality to behavioural motivation, has been a long-standing topic in the literature of human psychology (2–4). To maintain a sense of belonging, the individual must perceive their beliefs, lived experience and actions to be understood and accepted by group members; that is, the individual seeks *validation* by the social network.

The narratives of those with chronic pain suggest the need for their experience to be acknowledged, believed and supported (5–9). While the desire to feel validated is a clear theme expressed by individuals with pain, validation as a construct has yet to be defined adequately in the context of chronic pain. Thus, the primary aim of this paper was to build upon the current conceptualization of validation as applied in therapy, and as it appears thematically in pain narratives and other literature, to establish a substantive definition of *pain*-validation. Our second aim was to address the question “What are the psychosocial stressors impacting those with chronic pain?” This question was examined through the lens of Hobfoll's (10) conservation of resources theory of stress. A third aim was to identify the benefits of incorporating specific validation practices within pain management interventions, in terms of improving treatment motivation and adherence.

As pain is a subjective sensation, it is difficult to identify instances where an individual may be seeking secondary gains. This presents a clear shortfall for health practitioners who bear the responsibility for decisions regarding prescription of pain medications, declaring individuals to be fit or unfit for work, and supporting or rejecting insurance claims. The possibility of secondary gains may account for a degree of skepticism and invalidation of those with chronic pain. However, validating the patient's pain is still possible, irrespective of decisions regarding issues of medications or task fitness. In this paper we present strategies for incorporating validating approaches during pain consultations.

## Foundational themes of validation

Validation has been explored by specialists of “compassion in therapy” (11–13), with Leahy (12) describing validation as an acknowledgement of the individual's experience, and one that is recognized as reasonable and worthy of due attention. In her work with clients diagnosed with borderline personality disorder, Marsha Linehan espoused the importance of validation in early therapy sessions (14). Linehan (15, 16) submits that validation of the client requires the therapist to communicate acceptance and demonstrate that the client's expressions are worthy of attention, taking care not to discount or disparage. Linehan (16) likens her definition of validation to Rogers' (17, 18) “unconditional positive regard” in the therapy relationship. Rogers (17, 18) depicts unconditional positive regard as caring and non-judgemental acceptance of the client and their experience, and asserts that therapists must portray such an attitude for therapeutic change to occur.

Researchers have explored the types of communication styles used by medical practitioners, particularly relating to the validation and invalidation of the client during a consultation. In particular, patients with medically unexplained symptoms or contested illnesses, such as chronic fatigue syndrome, multiple chemical sensitivity (19) or fibromyalgia (20), have expressed difficulty having symptoms accepted as being legitimate by medical practitioners. As an alternative to closed and dismissive communication styles often experienced by patients with chronic pain (9, 21), Epstein et al. (22) describes a “partnering” communication style that seeks to understand the patient's experience, acknowledges uncertainty or ambiguity around symptoms, and welcomes patient input into the treatment plan.

Feeling validated, however, necessarily comes from the patient perspective. Thus, to define pain-validation, a clear understanding must come from the voices of those with chronic pain. In our previous work, we examined pain narrative literature using thematic analysis to identify themes representative of pain-validation and invalidation (23). A systematic search strategy (listed in the Appendix of our previous paper) (23) was applied to 5 databases in March 2019, resulting in a final collection of 431 articles suitable for analysis. Examination of the data corpus gave rise to 5 major themes, with narratives commonly expressing invalidation of pain as attitudes ranging from a *lack of belief* (7, 9) to a *lack of compassion* (5, 24), a *lack of understanding* by others (6), and *feeling stigmatized* (25, 26). Lack of validating one's own pain was identified in terms of guilt, shame, perceived moral failure and burdening of others, and was represented as the fifth theme of *critical self-judgement*.

These descriptions illustrate the primary need for individuals to have their experience confirmed as both understandable and deserving of empathy. Such conditions are foundational to normalizing thoughts, feelings, and actions of clients in therapy. By characterizing their feelings and behaviours as understandable, given the context and history of circumstances (12), and through the practice of self-compassion, barriers to healing such as client shame and self-criticism can be broken down (27). At its core, normalizing an individual's experience is about conveying that *it* and *they* are still acceptable, thereby providing assurance that the individual has not violated any terms of membership to the societal group.

## Defining pain-validation

This paper builds on the component themes of pain-validation identified in our comprehensive review of pain narratives (23) to offer a construct definition. These themes, together with additional discourse conveyed by therapists and leading researchers in the field (11, 12, 16, 18), suggest that the definition of pain-validation is grounded in one's communication of pain being acknowledged, deemed believable, and construed as acceptable. Thus, pain-validation incorporates three essential elements:

1. *Belief*: Pain-validation requires acknowledgment and belief that the experience is real and true for the individual. Pain is a construct that varies in severity, among other dimensions (e.g., sensation type and episode length). Furthermore, pain is subjective, and the meaning of its perception will be influenced by one's own conceptualization and definition of pain. With regard to pain-validation, the level of pain in absolute terms is of little importance; rather, it is the reported experience of the individual's suffering that matters. This can be eased by acknowledging its existence. In contrast, rejection of a person's claim or expression of experience acts to stonewall further communications, collaborative solutions and social support.
2. *Acceptability:* The term “acceptability” is used here to reflect one's values and attitudes toward the expressions of the individual with pain; that is, the degree to which those expressions are regarded as appropriate. Acceptability is used as a concept distinct from that of “pain acceptance”—a construct consisting of readiness to experience pain, and engagement in activities despite pain (28). Pain-validation requires that the individual's pain is deemed acceptable. It is agreed that the pain may arise from a combination of factors within the human body, and though the reasons for an individual's ongoing symptoms may sometimes be unclear, acceptability implies that the individual's suffering can be understood and empathized. Pain is endured by a substantial proportion of society and, to that extent, falls within the range of normal human experiences. Affirming another person's experience of pain as acceptable effectively normalizes that experience and, by extension, the individual, thus allowing them to maintain a secure position of group belonging.
3. *Communication to the individual*: Pain-validation by others necessitates a third feature in that the first two elements, belief and acceptability of the pain, are communicated to the individual, effectively removing doubts or perceptions of negative judgement.

In outlining this construct definition, it may be useful to distinguish here how pain-validation differs from the related construct of compassion. With its central components of belief and acceptability, pain-validation is an *attitude* communicated by the observer, with the specific intention of legitimising the sufferer's experience. Compassion, however, can be understood as a higher-order construct that refers to a *perspective* of shared humanity, whereby an individual becomes aware of, and emotionally moved by suffering (of the self or others), and feels inclined to alleviate it (29). While both constructs involve the witness and recognition of another's suffering, compassion includes an emphasis on intent to act to ease suffering (29), whilst the mainstay of pain-validation is in sanctioning the experience of pain, as communicated by the individual.

## Why do people invalidate pain?

Narrative literature is replete with stories of individuals who share a range of pain-invalidation experiences. A lack of *belief* regarding the sufferer's pain often begins with difficulties establishing medical evidence for symptoms. Indeed, there are numerous accounts of people who perceive a degree of pain-invalidation when visiting their healthcare professional (5, 7, 26). The biomedical model of pain regards pain as a direct result of tissue damage, and promotes that evidence must be present in connection with pain symptoms (30). However, diagnosis of a pain condition may be difficult for medical professionals where scans and tests fail to provide confirmation of injury or illness. Thus, medical professionals and insurance providers subscribing to the biomedical model, who cannot find evidence for a patient's pain, may regard their symptoms with skepticism (30) and refer the patient to a psychologist. While psychological intervention is recognized as an effective, evidence based approach for treating certain pain conditions (30), the inference by doctors that psychological issues are the underlying cause of their complaints can be invalidating for some patients, who may perceive that healthcare professionals have identified them as “hypochondriacs”, “faking,” or “crazy” (6, 7). *To avoid invalidating the patient's pain, it is important that doctors identify the link between emotional stress (psychological) and the subsequent stress response (biological) that may be exacerbating the patient's pain (physical)*.

The absence of medical evidence or lack of a doctor's diagnosis can, in turn, affect judgements made by the patient's wider social network, since Western society holds medical endorsement as key to verifying illness status (6). Moreover, employers, co-workers, friends and family may hold no better understanding than doctors about the characteristics and nuances of pain conditions and their symptoms. Fluctuating pain levels across the day or week can produce inconsistent patterns of activity or task capability, with symptoms virtually incapacitating the individual on 1 day, and abating the next (31). The variability in functional capacity may leave healthy individuals questioning the validity of the sufferer's claims (31).

The web of factors connecting pain and its by product, fatigue, are also unappreciated by many without ongoing pain. On one level, the experience of chronic pain can be physically exhausting and leaves the individual bereft of energy for engagement in valued and necessary activities. Pain can also interfere with the quality of sleep, causing additional fatigue throughout the day (32). Those without first-hand experience may be unaware of the draining nature of chronic pain and fail to make the connection between pain-related fatigue and the patient's current limitations around activities such as driving, socializing, or working at their former pace or load. Without a full understanding of pain and its constituent factors, others may invalidate pain, instead attributing the patient's performance failures to personal motives and character flaws such as hypochondria, attention-seeking, laziness, malingering, and commitment avoidance (30). A lack of visible evidence for an individual's pain symptoms, and a lack of understanding about the nature of pain, can undermine the credibility of the sufferer's claims and, thus, the *acceptability* of their pain.

Invalidation through unacceptability of pain can also occur at the level of the self and may be evidenced by discounting of one's own pain experience, or resisting a self-compassionate attitude, instead adopting a “toughen up” approach (33). Some feel guilty about even acknowledging their own discomfort, knowing of others with a potentially life-threatening condition (34). Guilt can extend through the individual's self-concept, seeing their illness as a burden to others who are left to take on additional duties or stress (35). Pain-invalidation by the self is also demonstrated as anger by those who regard pain as a failure of their own body (35). Invalidation by the self or by others may also relate to pre-formed attitudes toward pain, such as those who have been conditioned to view pain displays as self-indulgent or a sign of weakness. People can fear demonstrating compassion toward the self or others, having suffered abuse or rejection when showing vulnerability on previous occasions (27) and may, therefore, demonstrate less empathy and tolerance toward those expressing pain.

## The link between the stress-response and pain

When faced with challenge or stress, be it physical or psychological, the body responds by activating the hypothalamic pituitary adrenal (HPA) axis in an effort to make energy available, divert blood flow to muscles, and prepare physiological systems for fight or flight (36). Those with chronic pain may undergo prolonged periods of stress. As a result, persistent activation of the HPA axis may produce sustained, elevated levels of cortisol, causing detrimental effects such as the breakdown of cellular structures, fatigue, and compromised immune function (36). Additionally, stress triggers inflammatory processes and other bodily mechanisms that underpin chronic pain (37). Pain-validation is of great importance, therefore, since it is central in attending to, and alleviating stresses faced by those with chronic pain.

### Pain as resource stress

Given a human's inherent awareness that survival may hinge on maintaining their membership in the societal group, it is understandable that rejection by group members would cause stress. Lazarus and Folkman (38) purported that stress arises as a result of a perceived insufficiency of resources to cope with the challenges posed by one's environment, as appraised by the individual. Hobfoll (10) expanded on earlier stress theories, proposing the conservation of resources (COR) model which holds that stress arises from the threat to, or actual loss of resources, or from a lack of resource acquisition. Hobfoll's COR theory asserts that people work to build and maintain resources for coping with challenges. By Hobfoll's definition, resources consist of personal characteristics, objects, energies, and conditions that serve to increase the level of assets valued by the individual such as success, social status, and the accumulation of further resources. Thus, resources relevant to pain-coping include internal character traits such as self-esteem, optimism and goal pursuit; or other external conditions such as social support (10, 39, 40).

Further described within the COR model, Hobfoll (39) posited that resource *gain* becomes more important emotionally when the individual is faced with loss of resources. People with chronic pain may find it more difficult than most to acquire or retain personal resources. For example, individuals with pain conditions may experience a decline in functional mobility, employment capacity, optimism, and/or self-esteem (41). In the case of reduced employment, they may become financially depleted (8), a problem further compounded by the cost of medical treatment. Social status may decline with job loss as the individual forfeits the ranking associated with a particular job role; and personal status in the family home may be threatened if capacity as the main “breadwinner” or as the ideal “homemaker” is lost (42, 43). In addition, chronic pain is often accompanied by fatigue (32), hindering the individual's capacity to maintain social relationships that may otherwise serve as a support resource (43). For those with chronic pain, limited means of building a resource base, together with the cumulative taxing of current resources, creates major coping stresses under the paradigm of Hobfoll's (10) COR theory of stress.

The loss of resources through having pain comes, paradoxically, at a time when assets and reserves are needed more than ever. As other resources become depleted, people with chronic pain may rely on the social support of friends, family and community members. However, having one's pain invalidated indicates a denial of support (44), either moral or physical. As pain-validation incorporates belief and acceptability of reported experience, the invalidation of pain suggests that the individual's expressions of pain are, in fact, unacceptable. At best, the pain-invalidated person is left to manage alone in their suffering; at worst, the individual risks being ostracised by their social network if they maintain their claims of pain publicly.

### The stress of social exclusion

Anxiety is an ongoing psychological stress common to many living with long-term pain. Numerous aspects of chronic pain may cause anxiety, with a large range attributable to psychosocial conflicts, particularly those related to pain-invalidation. To the extent that pain communications are invalidated by societal others, continued expressions of pain constitute non-conformity to the values or standards of the societal group. Within the framework of exclusion theory, Baumeister and Tice (3) suggest that social exclusion may occur if an individual fails to contribute sufficiently toward benefiting the group. Thus, in accordance with exclusion theory, loss of functional ability and employability are undesirable qualities since they may be viewed as evidence of limited contribution capacity, and may therefore be a major source of anxiety for individuals with pain. Non-conformists to the group risk alienation and social exclusion which represents a fundamental threat to the self (3). Given the functional limitations inherent in many chronic conditions, patients with chronic pain may experience a heightened degree of anxiety over the prospect of denied social acceptance, since social support represents an important resource for coping (45, 46).

Exclusion from group membership may also occur, as posited by Baumeister and Tice (3), when individuals contravene the rules and standards of behaviour expected by the group, since rule-breaking threatens disruption to the harmony and living dynamic of the group. As such, all members must behave in accordance with the group's moral norms. For those whose pain remains unsanctioned, the receipt of financial benefits, specialized services, or exemption from standard commitments, may be viewed by many as acting outside of the conventions and rules expected of society members. As such, pain-invalidated individuals may bear the stress of being condemned by others in society who do not recognize theirs as a case for special allowances.

The physiological effects of social validation stress have been demonstrated by Shenk and Fruzzetti (47) in an experiment showing that individuals who received invalidating responses to their emotions arising from a stressful task demonstrated higher emotional reactivity, negative affect, heart rate, and skin conductance levels than individuals who were validated when expressing their feelings about the same task. Results supported the enhancing effect of validation on the individual's ability to regulate emotional reactivity in stressful conditions. Current research showing that social stress and negative emotions exacerbate pain perception (48) lends support to Shenk and Fruzzetti's (47) suggestion that individuals in validating environments may enjoy better health in the long term, since emotional support may be protective to those experiencing prolonged exposure to stress accompanying chronic pain (49). These findings are also supported from a physiological standpoint by studies showing that increases in cortisol levels and proinflammatory immune processes occur in response to perceived threats to the social self (50).

Several other experimental studies lend support to the concept of social influence on the perception of physical pain. Brown, Sheffield, Leary, and Robinson (51) found that participants exposed to social support during a cold pressor task reported lower levels of pain than participants who were denied social support during the same task. Furthermore, support for social influence on pain perception was demonstrated in fMRI studies (52) in that the experience of social exclusion used similar neural processing pathways to those typically recruited in processing physical pain.

**Figure 1:**
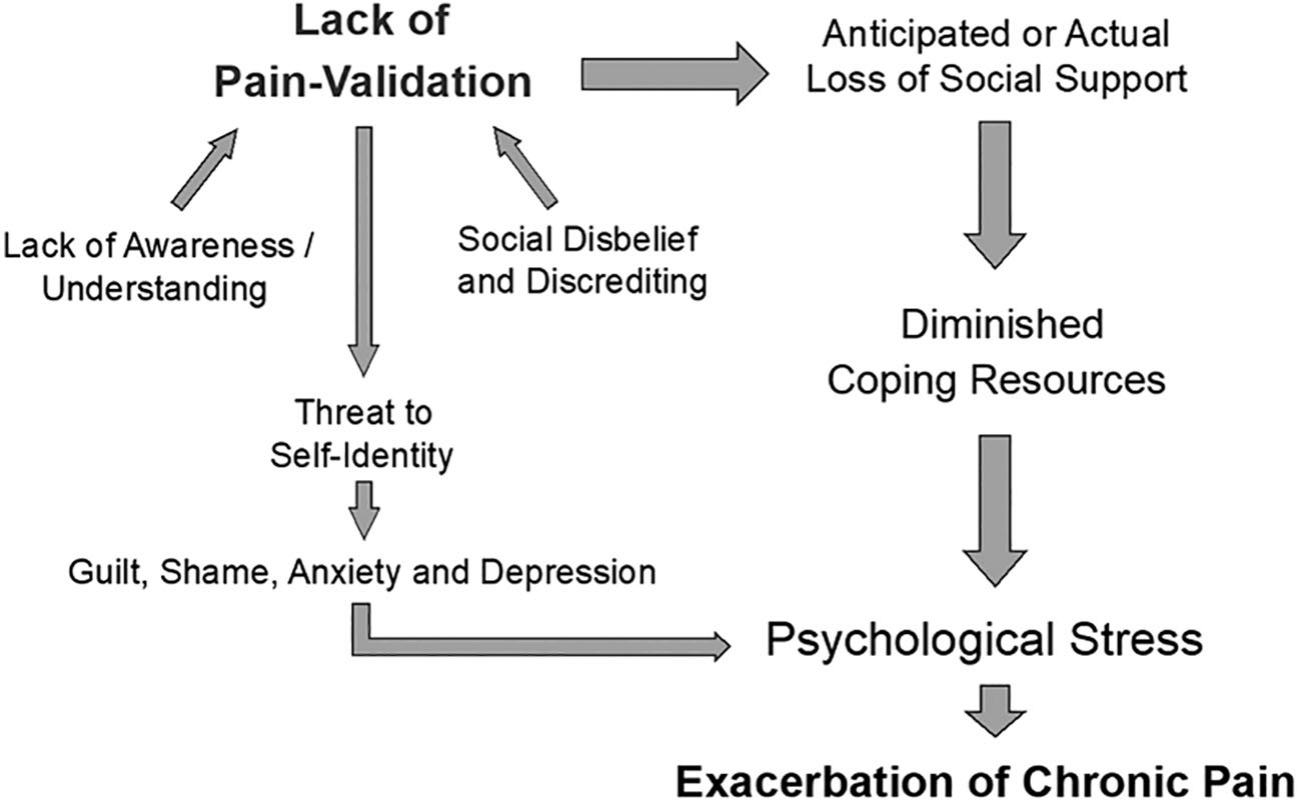
Proposed relationship between pain-invalidation, psychological stress, and the exacerbation of chronic pain. “Lack of Pain-Validation” is the overarching concept, consisting of lower-order components including “Lack of Awareness/Understanding” and “Social disbelief and discrediting”. *Lack of Awareness/Understanding* describes a failure to understand the nuances of pain conditions, and to recognise why medications may sometimes be ineffective. *Social disbelief and discrediting* refer to societal members' failure to believe that the patient has pain. Reasons may include an inability to establish medical evidence of a pain condition, the patient looks too well, and symptoms wax and wane. A lack of pain validation can promote further social discrediting and unacceptability. Ultimately, lack of pain validation may generate threats to self-identity and diminished coping resources which, in turn, exacerbate psychological stress and chronic pain.

Conversely, support for the role of social connectedness in moderating stress has been well documented (53, 54). Social support is associated with lower pain ratings and higher levels of pain-coping (55), and better functional capacity (45) in those with chronic pain. A possible explanation for these effects is offered in the findings that social support reduced neuroendocrine responses to social stressors, suggesting a lowered sensitivity to potentially stressful experiences (such as critical judgement) (56). Studies also show that the presence of oxytocin, a hormone associated with social bonding (57), attenuates levels of cortisol in response to social stress (58). Further studies showed that a combination of social support and oxytocin was associated with the greatest reduction in cortisol levels following social stress, when compared to participants in control conditions (no support or oxytocin), a group with only social support, and those administered oxytocin alone (58). Figure 1 illustrates how a lack of pain-validation and social support may relate to psychological distress and the potential exacerbation of chronic pain.

### The stress of identity loss

**Figure 2:**
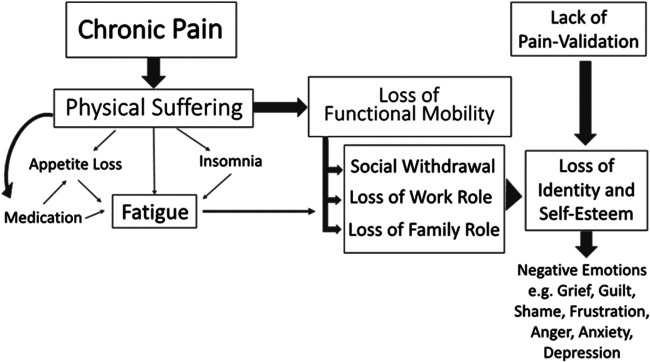
A holistic representation of chronic pain and commonly experienced consequences.

Beyond the stress imposed on sufferers who are socially unsupported, the importance of validation becomes apparent through a broader understanding of chronic pain and its consequent effects. Examination of narrative literature reveals that “having chronic pain” can be understood as more than the ongoing experience of physical discomfort. For many, having pain results in a myriad of direct consequences such as functional limitations which impede the capacity to work, to continue as the financial provider, perform home or family duties, and socialize with friends (43, 59). In these ways, living with chronic pain may result in a loss of identity, declining independence, reduced self-esteem, breakdown of relationships, and the resulting array of negative emotions (43, 59, 60). The outcomes of having chronic pain, as seen in Figure 2, may collectively be more problematic than the pain itself.

Chronic pain often prevents sufferers from behaving in ways that are consistent with the self-script, a script determined by their values. For example, people who value independence may struggle mentally with perceptions of incompetence and loss of self-worth in having to rely on others for help with shopping, looking after children, or even getting dressed, during a flare up of the condition (43, 61). In other instances, those who normally oppose drug use may battle internally with contravening their values to obtain pain relief *via* medications (62), and may potentially encounter external “drug abuser” stereotyping and stigma (25). For many, a substantial sense of identity loss ensues following work reduction or termination of their former career (60).

The changes and loss brought about by chronic pain interrupt the patient's view of their capabilities, identity, and understanding of the self in relation to the world (63, 64). Such losses or breakdown of self-script are often distressing, creating a destabilization of the sufferer's internal world and upsetting the stability of the psyche (65, 66), though the process is often noted as a precursor to the rebuilding of the self (63, 66). Humans rely on the consensus and verification of socially relevant others in forming perceptions about their own identity (67) and in establishing attitude norms (68). Social validation of the pain experience may help buffer pain-induced loss and destabilization by allowing for social communion, unity, and highlighting aspects of shared identity (69). The ability to tell one's story is also an important mechanism for re-organisation and formation of the new self following chronic illness, and helps the sufferer create meaning in the experience (64). Allowing opportunities for this narrative process, together with healthcare professionals' validation of uncertainty and loss experienced by their pain clients may, therefore, be beneficial (64, 70, 71).

## Proposed benefits of pain-validation in therapy

In addition to its many applications for stress reduction, pain-validation should be considered for its value in pain management interventions. Current therapies often incorporate, to varying degrees, validation of painful experiences (27, 72). However, academic literature is yet to explicitly identify pain-validation as it appears in the therapeutic context. Elements of pain management therapies that constitute pain-validation include developing the therapeutic alliance, psychoeducation, and pain education. Health professionals, broadly, may observe beneficial outcomes by purposefully incorporating such pain-validation techniques within the therapeutic encounter.

### Therapeutic alliance

One of the limiting factors to the efficacy of therapeutic interventions is the level of patient compliance or adherence (73). Poorer relationships with healthcare professionals relate to lower adherence to treatment plans (73), while healthy alliances relate to higher treatment adherence (74). This points to the benefits of developing rapport and a high-quality relationship between patient and professional, such that the patient feels heard and understood (74, 75). By validating pain through acknowledging the experience, and demonstrating belief in, and understanding of the patient, the therapist shows that (s)he is *mentalizing* the client's experience. Mentalization is an ability to understand the feelings, motivations, and behaviours of the self or others from their subjective perspective or mental state (76). In terms of chronic pain, mentalizing involves understanding the cognitions and emotions held by the patient regarding their pain, allowing for comprehension of the broad impact of pain and its consequences on the patient. Within the therapeutic relationship, mentalizing indicates to the patient that their experience is worthy of active consideration, enabling them to feel safe to discuss their pain and difficulties without fear of reprisal or judgement (75). Pain-validation is an active means of communicating mentalization, and is central to establishing patient trust in the therapist.

### Pain education and psychoeducation

Another way for the therapist to demonstrate their capacity to mentalize the patient's experience is by indicating an understanding of the deeper implications that having pain may hold for that patient. Those with chronic pain commonly feel a range of negative emotions including depression, fear, frustration and anger (77). Furthermore, experiences of injustice toward pain by social others are associated with adverse pain outcomes and resistance to change by individuals with pain (78). Pain-validation, then, offers a clear benefit in that it acts to create belief and acceptability, which can be demonstrated through the provision of pain education. For example, it may be helpful for the patient to hear that they are not alone in their suffering, nor abnormal in their condition, with many other people sharing similar symptoms or difficulties in achieving diagnoses. Rather than feeling defective, different and isolated, this form of validation allows the individual to maintain a sense of shared humanity, the importance of which is described as a core component of self-compassion (79).

A recently developed modality called Emotional Awareness and Expression Therapy (EAET) that gives focus to the role of emotional processing, has been successfully employed to reduce pain (80) and improve physical functioning (72). Therapists aim to educate patients about the role of stressful emotions in pain (71, 72). Patients are encouraged to face and express emotions that may have previously been unvoiced, particularly around trauma and conflicts, and techniques are taught to facilitate communication between the patient and close others (72).

Where appropriate, validation of pain symptoms may also be demonstrated by pain specialists providing pain education that offers alternative possible explanations for pain, particularly in the absence of scanning or laboratory-based evidence. Central sensitization is one such phenomenon theorised to account for continued stimulus sensitivity, and consequent pain signalling by neurons, even when injury and inflammation are no longer present (81). Sensitization may develop due to neural re-wiring at the site of injury, which may increase the excitability of neurons, or the number of neural synapses in the region (81, 82). Education about such mechanisms for pain is another core tenet of EAET with the aim of re-framing the patient's sense of pain as coming from the brain, and not necessarily due to further injurious movement (72). Learning about such possible reasons for otherwise unexplained pain may also provide validation regarding the “felt” experience, supporting patients who may have previously perceived practitioner judgements or disbelief regarding claims of pain.

Efforts made by the therapist to convey understanding of physical, psychological, and social difficulties (such as pain-invalidation in social circles) are fundamental to building the patient-provider relationship in the patient-centred approach (77). Sharing knowledge of general experiences reported in pain literature, potentially familiar to the patient, may provide further evidence of the therapist's ability to understand the complicated layers of distress associated with having a chronic condition. The therapist may choose to offer psychoeducation about the inability of social others to appreciate pain-related fatigue, the seemingly inconsistent appearance of pain symptoms in specific conditions, or the secondary effects of pain medications. This shared understanding may serve to enhance patient trust in the therapist and open the way for further communication and learning (75, 77).

Current pain management interventions often include modalities such as Cognitive Behavioural Therapy (CBT) and Acceptance and Commitment Therapy (ACT), that involve cognitive re-framing or behavioural modification and will, by definition, involve *change* (40). Re-framing and behavioural techniques are designed to arrest catastrophizing directions of thought, improve estimations of efficacy for pain-coping, increase general self-efficacy, build confidence for increasing functional mobility, and reduce pain-avoidance (30, 74). It is worth noting here that change may be difficult for several reasons. For example, both ACT and cognitive therapy techniques involve effortful and active practice of conscious awareness of thoughts and feelings, while remembering and applying newly learned strategies (83). Even more uncomfortably, change requires the mental exertion of pushing through boundaries of fear, deconstructing old realities, and disrupting maladaptive “scripts” or schemas, which once provided a sense of psychological stability (66). Hence, the prolonged effort and even discomfort necessary to instigate and adhere to change requires a degree of internal motivation. Such motivation may fail if the patient's fundamental struggle (having chronic pain) remains unacknowledged. Indeed, Linehan (16) warns of a client's need to feel validated prior to moving forward with interventions, underlining the problem that may occur if this need is unmet, whereby clients can remain “stuck” feeling invalidated, and resist treatment. Attempts to encourage change in clients at this stage may be experienced as dismissive of the client's feelings (16). Thus, pain-validation appears to be an important first step in pain management therapy by way of acknowledging the present experience of the client, and paying recognition to the difficulties in transitioning to life with chronic pain (70). Given the potential barriers to pain management interventions (84), the dual benefit of pain-validation in fortifying the therapeutic relationship and increasing patient motivation to embrace cognitive, behavioural, and affective change may go a long way toward improving treatment adherence (77, 84).

### Measuring pain-invalidation

Having established a construct definition of pain-validation, researchers can operationalize the construct and develop means of measuring levels of pain-validation or invalidation for those with chronic conditions. The Pain-Invalidation Scale (Pain-IS) is one such instrument, designed to measure pain-invalidation by the self, immediate others, healthcare professionals, and over-attending others (85). The Pain-IS enables the identification of areas where pain-invalidation may be limiting patient progress in therapy, or in functional rehabilitation. High scores in any domain may indicate the need to employ or teach validating communications as a first step in pain management therapy. Whether this increases compliance to interventions needs to be assessed.

## Conclusion

To date, pain-validation has remained a relatively abstract and undefined construct, and its importance in alleviating and protecting individuals from the stresses of chronic pain has not been comprehensively explored. We have attempted to address this shortfall by incorporating key elements of pain-validation into a definition, namely “communicating belief and acceptability of the sufferer's expressions of pain”. Pain was also explored in the context of Hobfoll's (10) COR theory of stress, with indications that much of the stress associated with having chronic pain relates to the potential loss of resources, such as financial losses, reduced connections with friends, lowered self-esteem, depleted physical capability, and lost status at home or work; as well as a reduced capacity for resource gain. Social support was recognized as an important resource for coping with chronic pain, as was the buffering effect of pain-validation against negative emotions. Importantly too, therapeutic modalities that teach pain-validation provide opportunities for emotional processing, with a view to reducing stress-related pain outcomes.

The potential value of pain-validation in therapeutic interventions was highlighted, bearing in mind that changes (in thinking or behaviour) require energy and motivation, which may be undermined by a failure to first acknowledge the current status of the individual. Pain management interventions may benefit from doctors and therapists identifying and discussing pain-invalidation experienced by the patient at the level of the self, in communications with healthcare professionals, and in their relationships with socially significant others. For example, explaining the link between stress and the possible exacerbation of pain may help patients feel validated and build trust within the doctor-patient relationship. Similarly, explaining the difference between validating and invalidating communications may help therapists teach effective partner communications. By providing a construct definition of pain-validation, we hope that researchers will further operationalize the construct to gauge levels of pain-validation and invalidation for those with chronic conditions.
